# Stressful Life Events and Neuroticism among Chinese Women with Recurrent Major Depressive Disorder

**DOI:** 10.62641/aep.v53i2.1730

**Published:** 2025-03-05

**Authors:** Fengfeng Chu, Chu Wang, Shufei Tao, Jingfang Gao, Xiangzhen Zhu, Danhong Tao, Bijun Chen, Ming Tao

**Affiliations:** ^1^The Second School of Clinical Medicine, Zhejiang Chinese Medical University, 310053 Hangzhou, Zhejiang, China; ^2^Department of Family Medicine, University of Virginia Medical Center, Charlottesville, VA 22903, USA; ^3^Psychiatry Department, The First Affiliated Hospital of Zhejiang Chinese Medical University, 310003 Hangzhou, Zhejiang, China; ^4^Clinical Psychology Department, The Second Affiliated Hospital of Zhejiang Chinese Medical University, 310005 Hangzhou, Zhejiang, China; ^5^Affiliated Mental Health Centre & Hangzhou Seventh People’s Hospital, Zhejiang University School of Medicine, 310013 Hangzhou, Zhejiang, China

**Keywords:** female patients, neuroticism, recurrent depression, stressful life events

## Abstract

**Background:**

Depression is associated with a high incidence of stressful life events (SLEs) and neuroticism. However, the impact of SLEs and neuroticism on the recurrence of major depressive disorder (MDD) remains unclear. Therefore, we aimed to identify the potential causal relationship between SLEs, neuroticism, and depression recurrence.

**Methods:**

This study included 5561 female patients with recurrent MDD (ages 30–60) and 4257 healthy volunteers (ages 40–60) from the China, Oxford, and Virginia Commonwealth University Experimental Research on Genetic Epidemiology (CONVERGE) study. We compared the female patients with recurrent MDD to a gender and age-matched group of healthy volunteers. Odds ratios (ORs) were calculated using logistic regression analysis to assess the impact of SLEs on depression onset. Furthermore, we employed bootstrapping sampling procedures to explore the mediating role of neuroticism between SLEs and the number of depressive episodes.

**Results:**

SLEs contributed to the occurrence of major depression, with rape (OR = 19.14, *p* = 0.004), serious neglect (OR = 3.65, *p* < 0.001), legal problems (OR = 2.51, *p* < 0.001), and divorce or relationship breakup (OR = 2.14, *p* < 0.001) being significantly associated with the onset of MDD. Following MDD onset, certain SLEs, such as the death of a family member (Z = 3.64, *p* < 0.001), unemployment (Z = 5.63, *p* < 0.001), job termination (Z = 6.43, *p* < 0.001), and financial crisis (Z = 5.53, *p* < 0.001), led to a significant increase in the number of depressive episodes. Furthermore, mediation analysis demonstrated that events such as divorce or relationship breakup (*p* < 0.05), rape (*p* < 0.05), financial crisis (*p* < 0.05), and physical abuse (*p* < 0.05) indirectly affected the number of depressive episodes through neuroticism.

**Conclusions:**

Our study demonstrates that SLEs in different categories have different effects on the onset and recurrence of MDD, and their effects regarding personal maltreatment, interpersonal relationship, and finance on the recurrence of depression are mediated by neuroticism.

## Introduction

Major depressive disorder (MDD) is the most common psychiatric disorder worldwide, characterized by manifestations such as depressed mood, loss of interest in activities, reduced energy level, and various somatic complaints. This condition significantly impacts psychological well-being, social behaviors, and overall quality of life, often contributing to suicidal tendencies [1, 2, 3]. Over the past 30 years, global MDD cases have surged by nearly 59%, afflicting more than 274.8 million people of all ages [4]. With a female preponderance and high recurrence rate, cross-cultural surveys have indicated that the 12-month prevalence of MDD is approximately 6% in the general population and the lifetime prevalence is 2–3 times higher than that [5, 6, 7].

The etiology of MDD is complex and diverse. Previous investigations have revealed that social and environmental adversities, especially stressful life events (SLEs), adversely impact both physiological and psychological health [8, 9, 10], playing a critical role in the occurrence, persistence, and recurrence of depression. Several studies have reported a strong link between SLEs and depression [11, 12, 13], suggesting that these events are closely linked with the initial onset of MDD compared to the subsequent episodes [14, 15].

Stressful life events can result from an individual’s behavior (e.g., relationship breakup, unemployment) or circumstances beyond one’s control (e.g., natural disasters, accidents). Studies reveal that both kinds of life events, dependent and independent, contribute to the development of depression [8, 16]. However, the relative impact of these two types of events on MDD is inconsistent [16, 17] and may involve different psychological mechanisms and moderators [18]. Specifically, events related to interpersonal relationship and finance, such as the death of a family member, a breakup in a long-term relationship, financial crisis, and job loss, have been closely associated with the occurrence of depression [16, 19, 20]. Additionally, events related to personal maltreatment have demonstrated the strongest connections with MDD [21, 22].

The association between personality traits and depression is well documented [23, 24]. Neuroticism, characterized by a persistent tendency to perceive negative emotions and marked by vulnerability and poor stress-coping skills, substantially increases the risk of depressive symptoms and is considered one of the strongest predictors of depression [25, 26]. Individuals with high neuroticism are more sensitive to depression [27, 28], and this effect is further enhanced when neuroticism interacts with SLEs [29, 30, 31]. Furthermore, SLEs and neuroticism have been identified as prognostic indicators of depression recurrence [32, 33]. However, a comprehensive understanding of the relationship between SLEs, neuroticism, and the recurrence of depression is yet to be explored.

Based on previous surveys and studies [12, 21, 28], we hypothesized that: (1) MDD patients experience more SLEs compared to healthy volunteers; (2) SLEs and neuroticism contribute to the onset of MDD, with personal maltreatment, interpersonal relationship issues, and finance-related events having a more pronounced effect than other type of events; (3) SLEs and neuroticism play a crucial role in the recurrence of MDD, with neuroticism mediating the relationship between SLEs and MDD recurrence. Therefore, to test these hypotheses, we administered a stressful life event questionnaire covering 16 categories of events and a neuroticism inventory to women with recurrent MDD and gender and age-matched healthy volunteers.

## Methods

### Study Participants

The data for this study were derived from the China, Oxford, and Virginia Commonwealth University Experimental Research on Genetic Epidemiology (CONVERGE) study on MDD, conducted between 2015 and 2016 [34]. We included 5561 patients with recurrent major depression, classified as MDD-total (mean age 43.91 years ± 8.74 S.D., age range 30–60). These patients were recruited from psychiatric departments of provincial mental health centers and general hospitals across 41 cities in 19 provinces and four central cities in China (i.e., Beijing, Shanghai, Tianjin, and Chongqing). Additionally, 4257 healthy volunteers (mean age 48.98 ± 5.43 years, age range 40–60 years) were recruited from patients undergoing minor surgical procedures at general hospitals or local community centers. All participants were Han Chinese women with four Han Chinese grandparents.

The inclusion criteria for patients included individuals aged between 30 and 60 years, patients who experienced at least two episodes of major depression meeting the Diagnostic and Statistical Manual of Mental Disorders-IV (DSM-IV) criteria [35], with the first episode occurring between 14 and 50 years, and had no history of drug or alcohol abuse before the onset of major depression. However, healthy volunteers were collected from the same region as the patients, aged between 40 and 60 years, had never experienced an episode of major depression, and had no blood lineage with the MDD patients. We opted the higher minimum age for healthy volunteers to reduce the chance of a late onset of major depression. Moreover, participants having other confounding factors, such as bipolar disorder, mental retardation, or any form of psychosis, were excluded from the study. The study protocol was approved by the Ethical Review Board of Oxford University and the ethics committee of the participating hospitals in China, and written informed consent was obtained from all participants.

Since the number of SLEs tends to increase with age [36, 37], a subgroup of patients, matched in age with healthy volunteers (i.e., age range 40–60) was established, called MDD-sub (n = 3594, mean age 49.19 ± 5.87 years). These two groups were used to compare SLEs and neuroticism between MDD patients and healthy volunteers and to examine the impact of SLEs and neuroticism on the onset of depression. Additionally, to assess the effect of SLEs and neuroticism on depression recurrence, data from all enrolled patients (MDD-total) were used.

### Assessment Measures

The diagnosis of MDD was determined using the Composite International Diagnostic Interview (CIDI) (WHO lifetime version 2.1; Chinese version), classified by experienced psychiatrists based on the DSM-IV criteria [33]. All participants were observed through a computerized assessment system developed by Oxford. The initial translation of the interview into Mandarin was performed by a team of psychiatrists at the Shanghai Mental Health Center and later reviewed and adjusted by the CONVERGE team. Both healthy volunteers and MDD patients underwent psychopathological and psychosocial functioning assessments, and their demographic and personal characteristics were recorded. The demographic factors assessed in this study included current age, age of first depressive episode, education level, occupation, social class, and marital status.

The SLEs questionnaire determines the occurrence of 16 traumatic life events and the age at their first occurrence, including the death of a family member, divorce or relationship breakup, unemployment, job termination, financial crisis, legal problems, serious illness, life-threatening accident, natural disasters, witness to someone injured, being raped, physical assault, physical abuse, severe neglect, being threatened, and other terrible events (covered in the 16th category of the questionnaire). This questionnaire was initially developed for the Virginia Adult Twin Study of Psychiatric and Substance Use Disorders [38] and has been used in a previous study [21].

The Eysenck Neuroticism Scale consists of 23 dichotomous items (yes/no) that measure the neuroticism trait, with more positive responses indicating a higher tendency towards neuroticism [39]. To minimize the impact of age on the findings, the neuroticism scores were normalized using a conversion table that correlates original and standard neuroticism scores in Chinese women. Moreover, these normalized scores were then used for further analyses.

### Statistical Analyses

Continuous variables that did not follow a normal distribution were presented as median (interquartile range), while mean and standard error values were used for other continuous variables. The Wilcoxon rank-sum test was used to evaluate the difference in the number of SLEs and neuroticism scores between the MDD-sub and healthy volunteers. However, the association between SLEs, neuroticism, and the onset of depression was assessed by employing logistic regression. The Wilcoxon rank-sum test was also utilized to observe the impact of SLEs occurring after depression onset on the number of depressive episodes in MDD-total. Linear regression was applied to analyze the relationship between SLEs, neuroticism, and episode numbers in MDD-total. The Chi-square test and multivariate logistic regression analysis were used to explore the impact of SLEs on depression onset in both healthy volunteers and MDD-sub. Considering neuroticism as a mediator, the bootstrapping sampling procedure (SPSS version 25.0, SPSS Inc., Chicago, IL, USA) was used to assess the indirect effects [40] between SLEs and the number of depression episodes. A *p*-value < 0.05 was considered statistically significant.

## Results

### Scale Scores

Compared to healthy volunteers (Table 1), the MDD-sub experienced more SLEs [1.00 (0.00, 2.00) vs. 1.00 (0.00, 1.00), *Z* = 18.04, *p* < 0.001] and had higher neuroticism scores [55.00 (45.00, 60.00) vs. 35.00 (25.00, 40.00), *Z* = 59.17, *p* < 0.001]. The frequency of SLEs in healthy volunteers, MDD-sub group, and MDD-total group is shown in Table 2.

**Table 1.** **The number of stressful life events (SLEs) and neuroticism scores between healthy volunteers (HV) and patients with recurrent major depressive disorder (MDD-sub and MDD-total)**.

Groups	Number of SLEs	HV vs. MDD-sub		HV vs. MDD-total		Neuroticism scores	HV vs. MDD-sub		HV vs. MDD-total	
		Z	*p*	Z	*p*		Z	*p*	Z	*p*
HV	1.00 (0.00, 1.00)	18.04	0.000	21.00	0.000	35.00 (25.00, 40.00)	59.17	0.000	66.74	0.000
MDD-sub	1.00 (0.00, 2.00)					55.00 (45.00, 60.00)				
MDD-total	1.00 (0.00, 2.00)					55.00 (45.00, 60.00)				

Notes: HV, healthy volunteers; MDD-sub, a subgroup of patients with major depressive disorder; MDD-total, patients with depressive disorder.

**Table 2.** **The frequency of stressful life events (SLEs) in healthy volunteers (HV) and patients with recurrent major depressive disorder (MDD-sub and MDD-total)**.

	HV (n = 4257)		MDD-sub (n = 3594)		MDD-total (n = 5561)	
Number of SLEs	N	%	N	%	N	%
0	2087	49.03	1151	32.03	1754	31.54
1	1156	27.16	1014	28.21	1552	27.91
2	594	13.95	676	18.81	1011	18.18
3	259	6.08	368	10.24	556	10.00
4	100	2.35	220	6.12	361	6.49
5	34	0.80	84	2.34	153	2.75
6	20	0.47	45	1.25	87	1.56
≥7	7	0.16	36	1.00	87	1.56

Notes: HV, healthy volunteers; MDD-sub, a subgroup of patients with major depressive disorder; MDD-total, patients with depressive disorder; N, the number of participants reported in each number of SLEs; %, percentage.

### Effect of SLEs on Depression Onset

After adjusting for educational level, occupation, social class, marital status, and current age, the number of SLEs was significantly associated with depression onset (odds ratio (OR) = 1.42, *p* < 0.001, 95% confidence interval (CI) = 1.36–1.47) (Table 3). Chi-square test analysis showed that 16 categories of SLEs, except for “witness to someone injured” and “natural disasters”, were associated with the incidence of MDD (Table 4). Furthermore, stepwise multivariate logistic regression analysis identified that 11 of these SLEs categories were positively correlated with the onset of MDD (Table 5). In contrast, the other 3 categories were not included in the model. Among these, the odds ratios (ORs) for being raped (OR = 19.14), severe neglect (OR = 3.65), legal problems (OR = 2.51), and divorce/relationship breakup (OR = 2.14) were substantially higher compared to other SLEs (ORs <2.00).

**Table 3.** **The impact of the number of SLEs on depression onset in healthy volunteers (HV) and age-matched female patients with recurrent major depressive disorder (MDD-sub)**.

Variables	B	S.E.	Wald	df	*p*	OR	95% CI for OR	
							Lower	Upper
Constant	–0.94	0.27	11.66	1.00	0.00	0.39		
The number of SLEs	0.35	0.02	288.69	1.00	0.00	1.42	1.36	1.47

Notes: B, partial regression coefficient; S.E., standard error; Wald, Wald Chi-Square Test; df, degrees of freedom; OR, odds ratio; 95% CI, 95% confidence interval.

**Table 4.** **The relationship of 16 stressful life events on depression onset in healthy volunteers (HV) and age-matched female patients with recurrent major depressive disorder (MDD-sub)**.

Stressful life events	HV	MDD-sub	Chi-square test analysis	
			χ ^2^	*p*
No death of a family member	3616	2675	135.42	0.000
Death of a family member	638	916		
No divorce/relationship breakup	3934	2967	178.64	0.000
Divorce/relationship breakup	320	624		
No unemployment	3830	3168	6.64	0.010
Unemployment	424	423		
No job termination	4141	3392	43.11	0.000
Job termination	113	200		
No financial crisis	3717	2919	55.79	0.000
Financial crisis	537	673		
No legal problems	4212	3465	60.94	0.000
Legal problems	42	128		
No serious illness	3925	3122	60.91	0.000
Serious illness	330	471		
No life-threatening accident	3981	3076	7.98	0.005
Life-threatening accident	274	272		
No natural disasters	3809	2964	1.80	0.180
Natural disasters	447	384		
No witness to someone injured	3945	3077	1.64	0.200
Witness to someone injured	311	271		
No being raped	4254	3305	48.77	0.000
Being raped	2	43		
No physical assault	4133	3128	58.95	0.000
Physical assault	123	220		
No physical abuse	4216	3220	72.11	0.000
Physical abuse	40	128		
No serious neglect	4170	3064	169.04	0.000
Serious neglect	86	284		
No being threatened	4245	3309	23.57	0.000
Being threatened	11	39		
No other terrible events	4088	3343	35.02	0.000
Other terrible events	168	250		

Notes: Due to missing data for some individual patients, the sum of data for the variable groups does not match n = 4257 for the HV group and n = 3594 for the MDD-sub group. HV, healthy volunteers; MDD-sub, a subgroup of patients with major depressive disorder; χ^2^, chi-square value.

**Table 5.** **The impact of 16 stressful life events on depression onset in healthy volunteers (HV) and age-matched female patients with recurrent major depressive disorder (MDD-sub)**.

Stressful life events	B	S.E.	Wald	df	*p*	OR	95% CI for OR	
							Lower	Upper
Constant	–0.91	0.28	10.40	1.00	0.001	0.40	-	-
Death of a family member	0.48	0.07	49.09	1.00	0.000	1.61	1.41	1.84
Divorce/relationship breakup	0.76	0.08	80.95	1.00	0.000	2.14	1.81	2.52
Unemployment	-	-	-	-	-	-	-	-
Job termination	0.65	0.14	21.01	1.00	0.000	1.91	1.45	2.52
Financial crisis	0.20	0.08	6.61	1.00	0.010	1.22	1.05	1.41
Legal problems	0.92	0.21	19.47	1.00	0.000	2.51	1.67	3.78
Serious illness	0.44	0.09	23.05	1.00	0.000	1.55	1.29	1.85
Life-threatening accident	-	-	-	-	-	-	-	-
Being raped	2.95	1.03	8.26	1.00	0.004	19.14	2.56	143.33
Physical assault	0.36	0.14	6.87	1.00	0.009	1.43	1.10	1.87
Physical abuse	0.68	0.24	8.17	1.00	0.004	1.97	1.24	3.15
Serious neglect	1.29	0.15	71.67	1.00	0.000	3.65	2.70	4.92
Being threatened	-	-	-	-	-	-	-	-
Other terrible events	0.36	0.13	8.19	1.00	0.004	1.43	1.12	1.84

Notes: B, partial regression coefficient; S.E., standard error; Wald, Wald Chi-Square Test; df, degrees of freedom; OR, odds ratio; 95% CI, 95% confidence interval.

### SLEs after MDD Onset and Depression Recurrence

In the MDD-total group, specific SLEs such as the death of a family member (Z = 3.64, *p* < 0.001), divorce or relationship breakup (Z = 3.49, *p* < 0.001), unemployment (Z = 5.63, *p* < 0.001), job termination (Z = 6.43, *p* < 0.001), financial crisis (Z = 5.53, *p* < 0.001), serious illness (Z = 2.62, *p* = 0.009), life-threatening accident (Z = 3.05, *p* = 0.002) that occurred after MDD onset resulted in an increase in the number of depressive episodes compared to those occurring before MDD onset (Table 6).

**Table 6.** **The association of stressful life events (SLEs) that occurred after major depressive disorder (MDD) onset to episode numbers of depression in all female patients with recurrent major depressive disorder (MDD-total)**.

Stressful life events	SLEs before MDD onset		SLEs after MDD onset		Z	*p*
	N	Episode numbers	N	Episode numbers		
1. Death of a family member	612	3.00 (2.00, 4.00)	336	3.00 (2.00, 4.00)	3.64	0.000
2. Divorce/relationship breakup	479	3.00 (2.00, 4.00)	434	3.00 (2.00, 4.00)	3.49	0.000
3. Unemployment	488	2.00 (2.00, 3.00)	299	3.00 (2.00, 4.00)	5.63	0.000
4. Job termination	208	2.00 (2.00, 3.00)	165	3.00 (2.00, 4.00)	6.43	0.000
5. Financial crisis	634	2.00 (2.00, 3.00)	320	3.00 (2.00, 4.00)	5.53	0.000
6. Legal problems	89	3.00 (2.00, 4.00)	99	3.00 (2.00, 3.00)	0.13	0.893
7. Serious illness	418	3.00 (2.00, 3.00)	152	3.00 (2.00, 4.00)	2.62	0.009
8. Life-threatening accident	292	2.50 (2.00, 3.00)	116	3.00 (2.00, 4.00)	3.05	0.002
9. Natural disasters	412	3.00 (2.00, 3.75)	107	3.00 (2.00, 4.00)	1.57	0.115
10. Witness to someone injured	276	3.00 (2.00, 4.00)	130	3.00 (2.00, 4.00)	1.68	0.094
11. Being raped	77	3.00 (2.00, 5.00)	16	3.50 (2.00, 5.57)	0.60	0.549
12. Physical assault	242	3.00 (2.00, 4.00)	107	3.00 (2.00, 4.00)	0.67	0.504
13. Physical abuse	213	3.00 (2.00, 4.00)	0	-	-	-
14. Serious neglect	492	3.00 (2.00, 4.00)	0	-	-	-
15. Being threatened	56	2.00 (2.00, 3.00)	18	2.50 (2.00, 5.00)	1.14	0.256
16. Other terrible events	332	3.00 (2.00, 4.00)	57	2.00 (2.00, 3.00)	0.58	0.562

Notes: N, the number of participants in each event.

### The Indirect Effect of Neuroticism between SLEs and Depression Recurrence

In the MDD-total group, the number of SLEs (β = 0.05, B = 0.08, standard error = 0.02, *p* < 0.001) and neuroticism scores (β = 0.09, B = 0.02, standard error = 0.003, *p* < 0.001) positively predicted the number of depressive episodes (adjusted R^2^ = 0.02) after controlling for demographic factors (Table 7). Moreover, neuroticism served as a mediator between certain categories of SLEs and the number of depressive episodes (Table 8). In the mediation model, with neuroticism as the mediator, the incidence of divorce or relationship breakup and being raped showed both direct effects (the events per se to episodes) and indirect effects (the events to neuroticism then to episodes) on increasing the number of depressive episodes. In contrast, financial crises, physical abuse, and other terrible events showed no direct effect. They only increased the number of depressive episodes through the mediation of neuroticism in these models.

**Table 7.** **The effect of neuroticism and the number of stressful life events on episode numbers of depression in all female patients with recurrent major depressive disorder (MDD-total)**.

	B	Standard error	β	*t*	*p*	95% CI for B	
						Lower	Upper
Number of SLEs	0.08	0.02	0.05	3.57	0.000	0.04	0.13
Neuroticism	0.02	0.003	0.09	6.21	0.000	0.01	0.03
Adjusted R^2^	0.02						

Notes: B, unstandardized coefficients; β, standardized coefficients; 95% CI, 95% confidence interval.

**Table 8.** **The indirect effect of neuroticism between stressful life events and episode numbers of depression in all female patients with recurrent major depressive disorder (MDD-total)**.

The path of mediation	Effect	Bootstrap 95% CI
Divorce/relationship breakup → neuroticism → episode numbers	0.08*	0.05–0.12
Divorce/relationship breakup → episode numbers	0.29*	0.09–0.48
Financial crisis → neuroticism → episode numbers	0.10*	0.06–0.13
Financial crisis → episode numbers	0.16	–0.03–0.35
Being raped → neuroticism → episode numbers	0.17*	0.11–0.24
Being raped → episode numbers	0.78*	0.23–1.33
Physical abuse → neuroticism → episode numbers	0.15*	0.10–0.21
Physical abuse → episode numbers	0.37	–0.003–0.73
Other terrible events → neuroticism → episode numbers	0.09*	0.06–0.13
Other terrible events → episode numbers	0.20	–0.08–0.48

Notes: *, indicates a significant effect in the path; 95% CI, 95% confidence interval.

## Discussion

This study aimed to elucidate the impact of SLEs, neuroticism, and their interaction on depression recurrence. Our analyses indicated that patients with MDD experienced more SLEs than healthy volunteers. SLEs related to personal maltreatment, such as being raped, severe neglect, physical abuse, and being threatened, significantly increased the risk of MDD onset, supporting our first two hypotheses. Our third hypothesis was supported by the following results: SLEs, including the death of a family member, divorce or relationship breakup, unemployment, job termination, and financial crisis that occurred after the onset of MDD, led to more episodes of depression. The number of SLEs and neuroticism scores predicted the number of depressive episodes. Considering neuroticism as a mediator, events such as divorce or relationship breakup, financial crisis, being raped, physical abuse, or other terrible events indirectly affected the number of depressive episodes.

The increased number of SLEs reported among MDD patients and the association between the number of SLEs and depression onset were consistent with findings from earlier studies [12, 41]. Previous reports [16, 42] have linked interpersonal relationship issues and finance-related events with an increased occurrence of depression. Our study further indicated that events related to personal maltreatment and legal issues, such as being raped, serious neglect, physical abuse, or legal problems, were particularly significant (with higher ORs) in the development of depression. Being raped, a severe subtype of sexual assault, can result in numerous adverse psychological consequences, such as post-traumatic stress disorder, depression, and anxiety [43, 44]. Childhood adversities, such as physical abuse and severe neglect, have been consistently correlated with the occurrence of depression [22, 45]. Additionally, legal problems, which can cause substantial psychological pressure, were also known to increase the risk of MDD [46].

The incidence of SLEs related to interpersonal relationship, employment, and finance occurred after MDD onset increased the number of depressive episodes compared to those occurred before MDD onset. While SLEs are known as significant risk factors for depression recurrence [47], not all SLEs display an identical impact in developing depression. Events that cause more damage to an individual’s sense of competence or social status tend to have a more profound impact on depression recurrence [48, 49]. Such events generally involve difficulties in interpersonal relationship, employment, and social status, such as the death of a loved one, breakup with a romantic partner, unemployment, and financial instability [8], all of which have been shown to exacerbate depression [50, 51, 52]. These observations support our results, indicating that the occurrence of such events after MDD onset increases the number of depressive episodes. Moreover, our findings can be explained in light of the stress generation hypothesis, which suggests a reciprocal relationship between depression and life stressors, leading to additional recurrences [53, 54, 55].

The findings of our study regarding the role of neuroticism in the onset and recurrence of MDD are consistent with previous investigations [27, 32, 56]. Furthermore, neuroticism was found to mediate the correlation between certain specific SLEs and the recurrence of depression. Individuals with high levels of neuroticism are more likely to divorce [57] and are more vulnerable to economic crises [58]. Similarly, physical abuse is positively correlated to neuroticism [59], and neuroticism has been shown to partially mediate the association between sexual abuse and depression [60, 61]. Collectively, neuroticism explains the relationship between these four categories of SLEs and the number of depressive episodes. Likewise, other terrible events, the 16th category in our Stressful Life Events questionnaire, also increased the number of depressive episodes through neuroticism. These findings suggest that future studies should include a more comprehensive categorization of stressful events.

The present study has several design limitations. Firstly, all participants were Chinese females, so further investigation involving all genders and different countries is needed before these results can be generalized to males or to other countries. Secondly, this study focused only on 16 categories of stressful life events. Including additional traumatic events and assessing the severity of their impact could offer a more comprehensive understanding of the effect of SLEs on depression. Thirdly, the measurement of SLEs, neuroticism, and depressive experience may be influenced by recall bias. Fourth, other factors such as extraversion, rumination, and perceived social support also play crucial roles in the development of depression. Future research should comprehensively consider these factors to better understand the relationship between life events and the onset of depression. Despite these limitations, this study demonstrates that SLEs and neuroticism are correlated with both the onset and recurrence of MDD. Personal maltreatment events preceding depression onset and interpersonal relationship, employment, or finance-related events occurring after depression onset were crucial in recurrent MDD. Neuroticism acted as a mediator in certain categories of events contributing to depression recurrence, helping to explain the association between SLEs and depression recurrence. These findings enhance our understanding of depression’s etiopathology and may offer new perspectives for preventing and treating recurrent depression.

## Conclusions

In the current study, we found that MDD patients experienced more SLEs, and those related to personal maltreatment, interpersonal relations, employment, and finance contributed to the onset and recurrence of MDD. We also found that with neuroticism as a mediating variable, SLEs indirectly influenced the number of depressive episodes. These observations demonstrate the potential impact of SLEs and neuroticism on MDD and provide clinical evidence supporting the use of personality therapy in treating recurrent depression to alleviate depressive episodes.

## Availability of Data and Materials

The datasets used and/or analyzed during the current study are available from the corresponding author upon reasonable request.
